# Determination of the expression of three fimbrial subunit proteins in cultured *Trueperella pyogenes*

**DOI:** 10.1186/s13028-018-0407-3

**Published:** 2018-09-12

**Authors:** Mengcheng Liu, Bing Wang, Hongmin Liang, Bo Ma, Junwei Wang, Wenlong Zhang

**Affiliations:** 1 College of Veterinary Medicine, Northeast Agricultural University, Changjiang Road 600, Harbin, 150030 Heilongjiang People’s Republic of China; 2 Key Laboratory of Animal Pathogen Biology of Ministry of Agriculture and Rural Affairs of the People’s Republic of China, Northeastern Science Inspection Station, Harbin, 150030 Heilongjiang People’s Republic of China

**Keywords:** Expression, Fim A, Fim C and Fim E, *Trueperella pyogenes*, Virulence factors

## Abstract

**Background:**

*Trueperella pyogenes* is a commensal and a significant opportunistic pathogen in animals. A variety of identified or putative virulence factors are considered to significantly contribute to the occurrence of *T. pyogenes* infection in different species. However, these virulence factors are not fully understood.

**Results:**

In the current study, the genes encoding putative fimbrial proteins, i.e. Fim A, Fim C, and Fim E, were cloned. Recombinant Fim A (rFim A), Fim C (rFim C), and Fim E (rFim E) were prepared and used to generate rabbit anti-rFim A, anti-rFim C, and anti-rFim E serum, respectively. Using these sera, we found that only Fim E was constitutively expressed in *T. pyogenes*. The expression level of Fim E in *T. pyogenes* peaked within 6–10 h of culture period in pH 7.5. Fim E protein expression was unaffected by anaerobic condition, but was inhibited by the microaerophilic condition. Tube agglutination tests indicated that Fim E was exhibited on the surface of *T. pyogenes* cells because anti-rFim E serum caused strong agglutination. Additionally, the blots for Fim A detection showed nonspecific reactions. Furthermore, the tube agglutination tests showed that anti-Fim A serum failed to cause agglutination of *T. pyogenes* cells, which indicated that Fim A was not, or poorly, expressed in cultured *T. pyogenes*. Anti-rFim C serum caused strong agglutination. However, the blots for Fim C detection showed a strong nonspecific reaction. Thus, the expression of Fim C was difficult to be determined using the current method.

**Conclusions:**

Fim E was expressed in cultured *T. pyogenes.* However, Fim A was either not or poorly expressed in cultured *T. pyogenes*. Moreover, Fim C expression was not determined using the current strategy.

**Electronic supplementary material:**

The online version of this article (10.1186/s13028-018-0407-3) contains supplementary material, which is available to authorized users.

## Background

*Trueperella pyogenes* is a Gram-positive rod-shaped bacterium [1]. This organism is a commensal and a common opportunistic pathogen in different domestic and wild animals [2–4]. Moreover, human beings can be infected by this bacterium; however, the infections are generally related to occupational exposure [5].

The ability of the *T. pyogenes* in causing infections in different species could be, at least partially, attributed to various virulence factors that are encoded in its genome [1]. The virulence factors of this bacterium include exotoxins (pyolysin), exoenzymes (proteases and DNase), and adhesins [1]. Among these virulence factors, components that mediate the adhesion of the organism to host cells play essential roles in establishing the infection [6, 7]. For *T. pyogenes*, these host cell adhesive factors not only work in establishing the infection but also when the organism acts as a commensal on the various mucous membranes of the hosts. Thus, investigations have been conducted on adhesins to help understand the pathogenicity of *T. pyogenes* and its mechanism in terms of switching from a commensal to a pathogen. Many adhesive components, such as collagen-binding protein A, fibronectin-binding protein, and fimbriae, that might mediate the adhesion of this organism to the host tissue, have been reported [1]. The genes encoding these adhesive components have been extensively used as molecular markers for identifying, genotyping, or predicting the virulence of *T. pyogenes* [3, 8]. However, evidence of adhesin expression in *T. pyogenes* cells is extremely limited [9–11]. *T. pyogenes* has been reported to express fimbriae [1]; however, the properties and constitution of this structure have not been studied. Putative fimbrial subunit proteins, such as Fim A, Fim B, Fim C, Fim E, and Fim J, have been reported to be expressed in *T. pyogenes.* However, only the genes, mRNAs, or the existence of specific antibodies against these proteins in *T. pyogenes*-infected animals have been detected [1, 12, 13].

In the current study, we aimed to determine the expression of three putative fimbrial subunit proteins, namely, Fim A, Fim C, and Fim E, in cultured *T. pyogenes*. However, only the expression of Fim E was detected. These results will contribute to the work on molecular pathogenesis regarding *T. pyogenes*.

## Methods

### Bacterial culture

Single colonies of *T. pyogenes* strains 0912 and 1005 were obtained by inoculating the bacteria on Martin broth agar plates (AOBOX Biotechnology Co., Beijing, China) with 10% sheep blood. The isolates originated from the lung of cattle that had dies due to pneumonia in China. The agar plates with the inoculum were incubated at 37 °C for 48 h. For the enrichment culture of the bacteria, single colonies of each *T. pyogenes* strain were inoculated into liquid Martin broth medium (AOBOX Biotechnology Co., Beijing, China) with 10% fetal bovine serum (FBS) and shaken at 37 °C for 24 h. The cultures were adjusted to reach an optical density (OD) 600 nm value of 1.2 using fresh culture medium, and then inoculated into fresh culture medium at a ratio of 2%.

In some of the experiments, the pH of liquid Martin broth medium was adjusted to different end points, using HCl (1 mol/L) or NaOH (1 mol/L), as indicated in the results.

For the anaerobic culturing of the bacteria, liquid paraffin was used to seal the surface of the liquid Martin broth medium. After inoculation, a 48-h static culture was used for bacterial proliferation. Moreover, the bacteria were cultured in a microaerophilic environment. Briefly, the bacteria were inoculated into the Martin broth agar plates with 10% sheep blood. The plates were placed in a jar. AnaeroPack-MicroAero (Mitsubishi Gas Chemical Company, Tokyo, Japan) was used to generate a microaerophilic environment. After 24 h, the lawn from the culture was harvested by washing the plates with sterile phosphate buffer saline (PBS).

In most of the above experiments, bacterial growth was determined by measuring the OD 600 nm values of the cultures. The purity of the cultures was determined by using streak plate method and Gram staining.

### Cloning and expression of *fimA*, *fimC*, and *fimE* genes

The genome of the bacteria was extracted using a Bacterium genome extraction kit (Tiangen Biotech, Beijing, China) according to the manufacturer’s instruction. The genes encoding the Fim A, C, and E proteins were amplified and cloned using the primers listed in Table 1. The genes were inserted into a prokaryotic expression vector, pET-30a (+) (Novagen, Madison, USA) through the corresponding restriction sites. The transformants were sequenced by BGI International using a T7 promoter and a terminator primer. The recombinant plasmids were named pET-30a (+)-*fimA*, pET-30a (+)-*fimC*, and pET-30a (+)-*fimE*.

**Table 1:** Primers used for gene cloning and plasmid construction

Names of the primers	Nucleotide (5′ to 3′)	Restrict sites
*fimA* forward	CGAGCTCATGGGTGCACTGGTGACGGC	*Sac* I
*fimA* backward	CCCAAGCTTTTAGGCATTACGACGACGAGCGAC	*Hin*d III
*fimC* forward	CGCGGATCCGTGTTTACGGTGGTAACC	*Eco*R I
*fimC* backward	CCCAAGCTTTTAGCTACGACGACGAGCGTG	*Hin*d III
*fimE* forward	CGAGCTCATGAAGCGAAACAAGTTACGTGC	*Sac* I
*fimE* backward	CCCAAGCTTTTAGACGTCCTTGCGTCGGTT	*Hin*d III

Restriction enzyme recognition sites are underlined in each sequence and listed in the right column

All these recombinant plasmids were transformed into *Escherichia coli* Rosetta (DE3)™ competent cells (Novagen, Madison, USA). The positively transformed *E. coli* were cultured at 37 °C until they reached an OD 600 nm value of 0.6–0.8. Recombinant Fim A (rFim A), Fim C (rFim C), and Fim E (rFim E) were expressed by inducing with 1 mmol/L isopropyl-β-d-1-thiogalactoside (IPTG) for 4 h and were then purified using nickel-charged resin (Merck, Shanghai, China). The purified proteins were dialyzed against PBS with 5% glycerol at 4 °C for 48 h. The proteins were quantified using Bradford Protein Assay Kit (Beyotime, Beijing, China), according to the manufacturer’s instructions and stored at − 80 °C until use. The signal peptides of these proteins were predicted using DNAMAN (LynnonBiosoft, San Ramon, USA).

### Preparation of rabbit sera against *T. pyogenes*, Fim A, Fim C, and Fim E

*T. pyogenes* strain 1005 was cultured in 50 mL Martin broth medium with 10% FBS. The culture was harvested after being incubated at 37 °C for 20 h, and then inactivated by treating with 0.5% formaldehyde at 37 °C for 72 h. The inactivated culture was centrifuged and the precipitation was resuspended in 20 mL normal saline (0.9% NaCl w/v). Aluminum hydroxide gel suspension was prepared as described in a previous study [14]. The inactive *T. pyogenes* culture was mixed with aluminum hydroxide gel suspension at a volume ratio of 5:1.

The protocol of the animal experiment was approved by the Ethics Committee on the Use and Care of Animals, Northeast Agricultural University, China.

Rabbit anti-*T. pyogenes* strain 1005 serum was prepared through subcutaneous inoculation of a New Zealand rabbit with 1 mL mixture of inactive *T. pyogenes* (4.1 × 10^9^ cells) and aluminum hydroxide gel twice with an interval of 15 days. Seven days after the second inoculation, the rabbit was boosted with 1 mL live *T. pyogenes* (4.1 × 10^9^ cells). Seven days after the boosting, serum was collected from the rabbit and stored at − 20 °C.

Rabbit anti-rFim A, anti-rFim C, and anti-rFim E sera were prepared by immunizing New Zealand rabbits with the corresponding recombinants. Each recombinant protein was used to immunize one rabbit. Each rabbit received two inoculations at a 2-week interval. A mixture of 500 μg recombinant protein and Freund’s complete adjuvant (Sigma-Aldrich, Shanghai, China) was used for the first inoculation, and a mixture of 500 μg recombinant protein and Freund’s incomplete adjuvant (Sigma-Aldrich, Shanghai, China) was used for the second inoculation. Three days after the second immunization, serum was collected and stored at − 20 °C.

### Determination of the reactivity of recombinant proteins to anti-*T. pyogenes* serum

The reactivity of the three recombinant proteins to rabbit anti-*T. pyogenes* serum were determined by using western blot assay. Purified rFim A, rFim C, and rFim E (10 μg each) were separated via sodium dodecyl sulfate polyacrylamide gel electrophoresis (SDS-PAGE) and were transferred onto nitrocellulose (NC) membranes. The NC membranes were blocked with 10% skim milk in PBST (0.05% v/v Tween 20 in PBS) for 1 h at room temperature. The membranes were then washed with PBST 5 times. The anti-*T. pyogenes* serum was diluted (1:10,000) and incubated with the NC membranes at 4 °C overnight and washed with PBST five times. Horseradish peroxidase (HRP) conjunct goat anti-rabbit IgG (ZSGB-bio, Beijing, China) was diluted (1:5000) and incubated with the NC membranes for 1 h at room temperature.

To verify the presence of the three recombinant proteins used for the analysis, a parallel immunoblot assay was performed using anti-histidine tag monoclonal antibodies (1:5000) (ZSGB-bio, Beijing, China) and anti-mouse HRP conjunct secondary antibodies (1:5000) (ZSGB-bio, Beijing, China) that were incubated with the NC membranes at 4 °C overnight and for 1 h at room temperature.

### Determination of the expression of Fim A, Fim C, and Fim E in cultured *T. pyogenes*

The expression of Fim A, Fim C, and Fim E in cultured *T. pyogenes* were determined by using western blot assay. Briefly, the *T. pyogenes* strain 0912 and strain 1005 cultures were harvested, and the collected cultures were centrifuged at 4 °C. The precipitations were washed with sterile PBS twice. Moreover, the bacteria were resuspended in sterile PBS and ultrasonically broken down (500 W) for 10 min (5 s with intervals of 3 s). The products were centrifuged at 4 °C, and the supernatants were harvested for further analysis. The concentrations of the proteins in supernatants were determined using Bradford Protein Assay Kit (Beyotime, Beijing, China). Afterward, the samples were adjusted using the same concentration. A total of 40 μg of proteins in samples were separated through SDS-PAGE. The expression of Fim A, Fim C, and Fim E in the samples were determined using rabbit anti-rFim A (1:5000), rFim C (1:2000), and rFim E (1:2000) sera, respectively. The primary antibodies were incubated with the NC membranes at 4 °C overnight. HRP conjugated goat anti-rabbit IgG (ZSGB-bio, Beijing, China) was diluted (1:5000) and incubated with the NC membrane for 1 h at room temperature.

### Tube agglutination test

*T. pyogenes* strains 0912 and 1005 cells were cultured at 37 °C until the OD 600 nm value reached 1.75. The cultures, in volumes of 50 mL, were centrifuged at 4 °C. The supernatants were discarded and the precipitates were washed twice with sterile PBS. The final precipitations were resuspended in 25 mL sterile normal saline. The bacterial suspensions were used as antigens for the tube agglutination test.

Tube agglutination test was utilized to determine the reactivity of the anti-rFim A, anti-rFim C, and anti-rFim E sera to *T. pyogenes* cells. The twofold serially diluted sera by normal saline (0.5 mL) was mixed with equal volumes of bacterial suspension. The tubes were incubated at 37 °C for 30 min. Unimmunized rabbit serum was added in every set of the test as controls.

## Results

### Cloning *fimA*, *fimE*, and *fimC* genes of *T. pyogenes*

The *fimA*, *fimC*, and *fimE* genes were amplified from genomes of *T. pyogenes* strains 0912 and 1005. The *fimA*, *fimC*, and *fimE* genes from *T. pyogenes* strain 0912 were 1362, 1374, and 1755 base pair (bp), respectively. The *fimA* and *fimC* genes of *T. pyogenes* strain 1005 showed the same length as the genes from strain 0912, whereas three nucleotide (382–384) deletions were observed in the *fimE* gene from *T. pyogenes* strain 1005, in comparison with *fimE* gene from strain 0912. The homologies of *fimA*, *fimC*, and *fimE* genes from the two strains were 98.83%, 99.04%, and 96.75%, respectively. The homologies of the deduced primary structures of Fim A, Fim C, and Fim E protein from the two strains were 98.01%, 99.43%, and 96.23%, respectively.

The primary structures of putative Fim A, Fim C, and Fim E proteins of *T. pyogenes* strain 0912 were analyzed using DNAMAN, which predicted signal peptides with lengths of 22, 27, and 30 amino acids (aa) at the carboxyl terminals of the three proteins, respectively. As reported previously [12], the deduced primary structures of Fim A, Fim C and Fim E proteins possess a cell wall sorting signal (CWSS), which is composed of a LPLTG motif and located approximately 30 aa away from the C terminus of these proteins (amino acid sequences of the three putative proteins of *T. pyogenes* strain 0912 are provided in Additional file 1).

### Preparation and identification of rFim A, rFim C, and rFim E

**Fig. 1:**
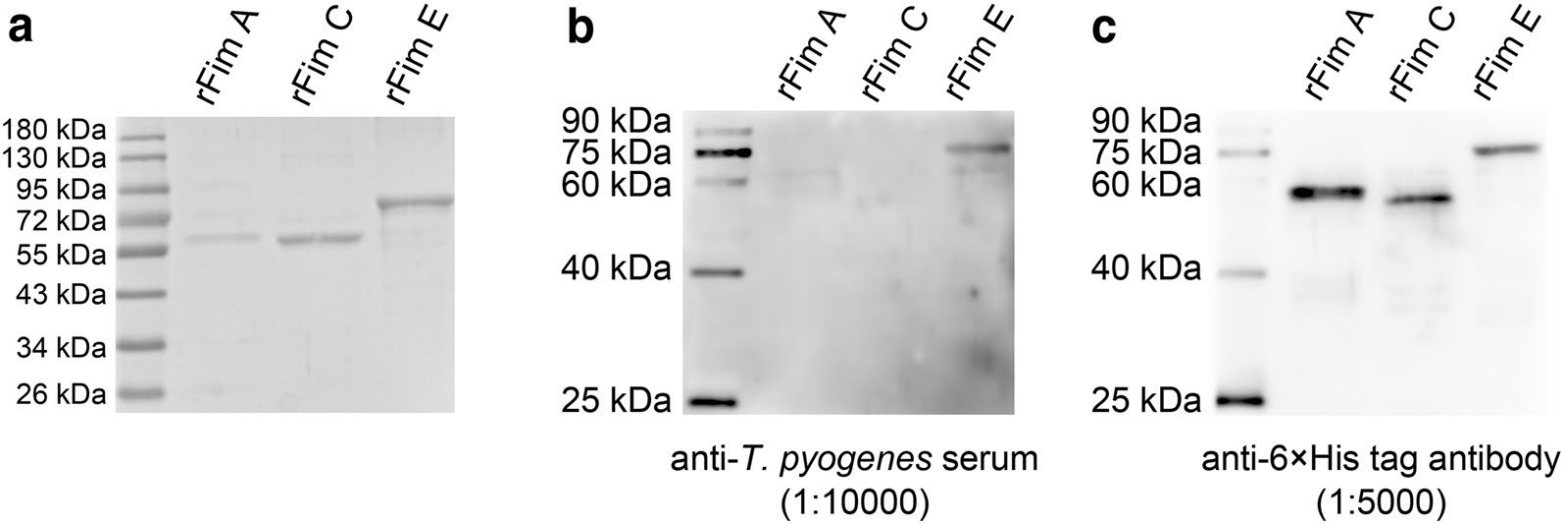
Analysis of the molecular weight and immunoreactivity of the rFim A, rFim C, and rFim E proteins by SDS-PAGE and western blot assays. **a** Purified rFim A, rFim C, and rFim E were analyzed via SDS-PAGE. The predicted molecular weight of rFim A, rFim C, and rFim E was 58 kDa, 58 kDa, and 72 kDa, respectively. **b** Immunoreactivity of rFim A, rFim C, and rFim E to rabbit anti-*T. pyogenes* serum. The serum was diluted 1:10,000. **c** Determination of the existence and the amount of rFim A, rFim C, and rFim E on the NC membrane via immunoblot assay using anti-histidine tag monoclonal antibody. The antibody was diluted 1:5000

As the target proteins from the two strains showed relatively high homology, only genes from *T. pyogenes* strain 0912 were expressed in *E. coli*. The rFim A and rFim C showed a molecular weight of approximately 58 kDa, whereas rFim E weighed approximately 72 kDa (Fig. 1a). Furthermore, western blot assay showed that rFim E, but not rFim A and rFim C, reacted with the anti-*T. pyogenes* serum (Fig. 1b). Results indicated that only anti-Fim E antibodies were elicited when the rabbit was immunized with *T. pyogenes*.

### Determination of the expression of Fim A, Fim C, and Fim E in cultured *T. pyogenes* strain 0912

**Fig. 2:**
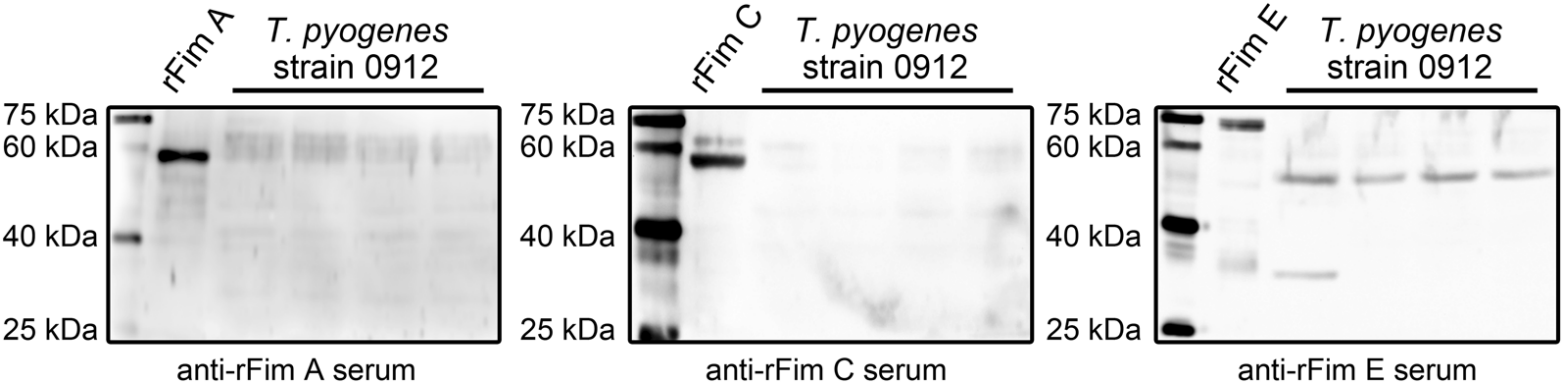
Analyzing the expression of Fim A, Fim C, and Fim E in cultured *T. pyogenes* strain 0912 by western blot assays. The samples for immunoblot assay were harvested from four different batches of cultured *T. pyogenes* strain 0912, all cultured for 4 h

*Trueperella pyogenes* strain 0912 was cultured for 4 h. The western blot assay indicated that only Fim E (predicted molecular weight was 56.6 kDa, without signal peptide and amino acids behind CWSS) was detected in the bacterium (Fig. 2). The blots for Fim A (predicted molecular weight is 42.2 kDa) and Fim C (predicted molecular weight was 41.1 kDa) detection showed nonspecific reaction bands. However, at the predicted area on the blots, none of the bands showed stronger reactivity than those of the other bands with the corresponding antiserum.

### Determination of the effects of different cultural conditions on the expression of Fim A, Fim C, and Fim E in *T. pyogenes* strains 0912 and 1005

**Fig. 3:**
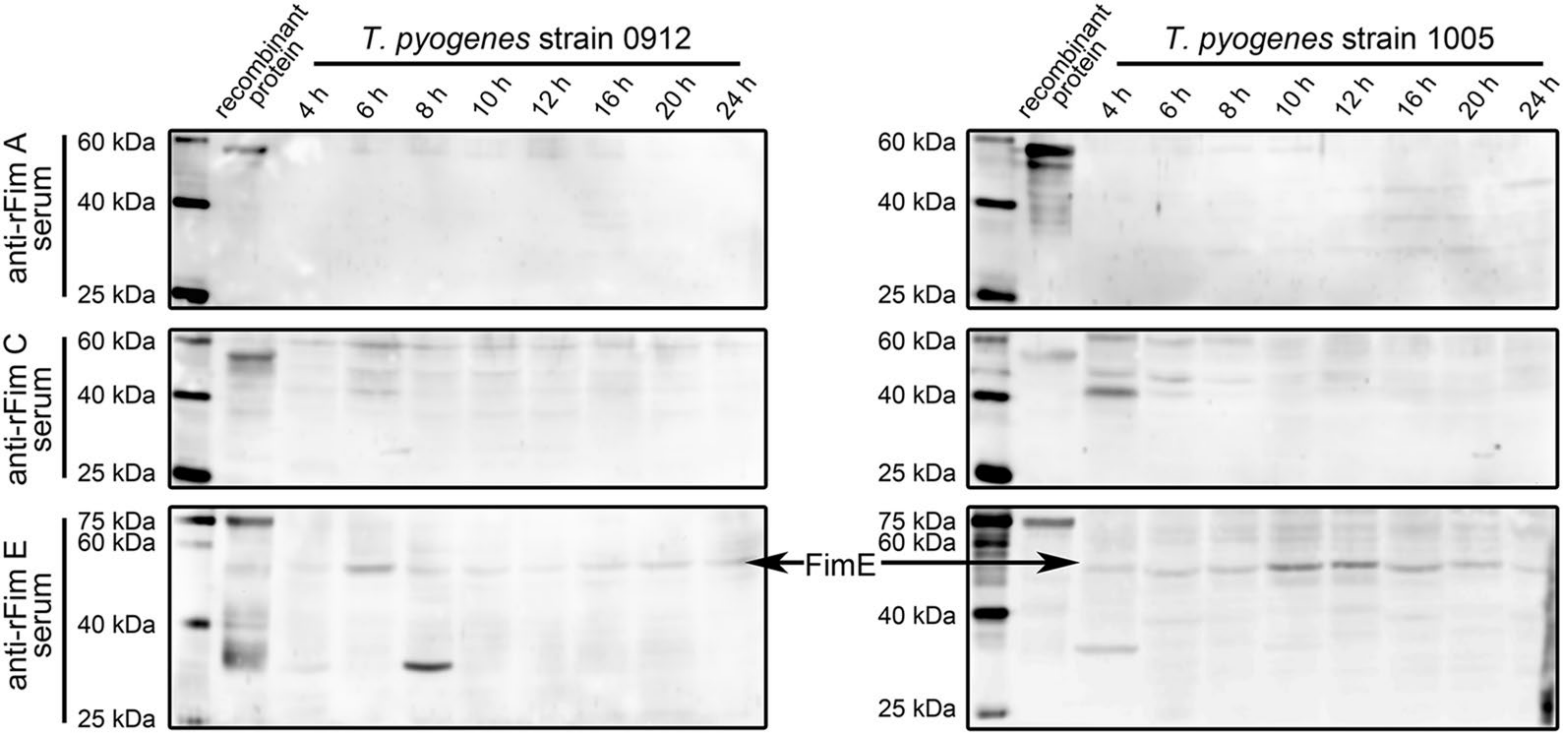
Analyzing the effect of culture period on the expression of Fim A, Fim C, and Fim E in *T. pyogenes* strain 0912 and strain 1005 by western blot assays. *T. pyogenes* strain 0912 and strain 1005 cells were cultured for 24 h. Samples were collected at 4, 6, 8, 10, 12, 16, 20, and 24 h. The experiment was repeated at least three times

We initially determined the influence of culture period on the expression of the three proteins. As shown in Fig. 3, we sampled *T. pyogenes* strains 0912 and 1005 cultures at 4, 6, 8, 10, 12, 16, 20, and 24 h post-inoculation. The expression of Fim E was observed in *T. pyogenes* strains 0912 and 1005 and was detectable in all samples collected at different time points. The expression of Fim E peaked at 6 h for strain 0912, and 10 h for strain 1005. The blots for Fim A and Fim C detection showed many nonspecific reaction bands.

**Fig. 4:**
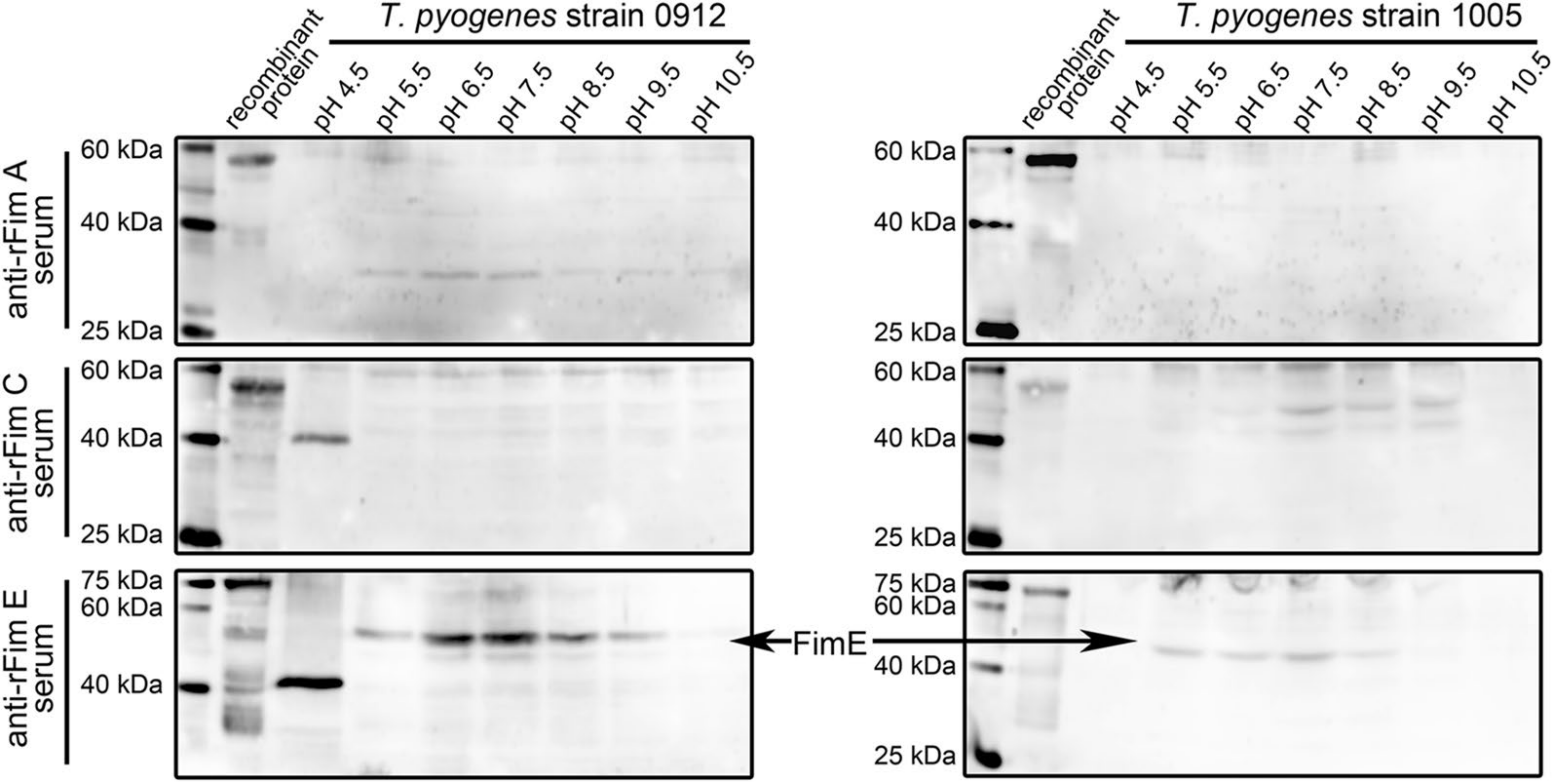
Analyzing the effect of pH value on the expression of Fim A, Fim C, and Fim E in *T. pyogenes* strain 0912 and strain 1005 by western blot assays. *T. pyogenes* strain 0912 and strain 1005 cells were cultured at pH 4.5, 5.5, 6.5, 7.5, 8.5, 9.5, and 10.5 conditions for 8 h. The experiment was repeated at least three times

We determined the influence of pH on the expression of the three proteins in *T. pyogenes* strains 0912 and 1005. Effective proliferation of *T. pyogenes* was observed at pH 6.5, 7.5, and 8.5 within 8 h and were partially inhibited at pH 4.5, 5.5, 9.5, and 10.5. Figure 4 show that Fim E expression could be observed in organisms cultured at pH 5.5–8.5. The blots for Fim A and Fim C detection still showed many nonspecific reaction bands.

**Fig. 5:**
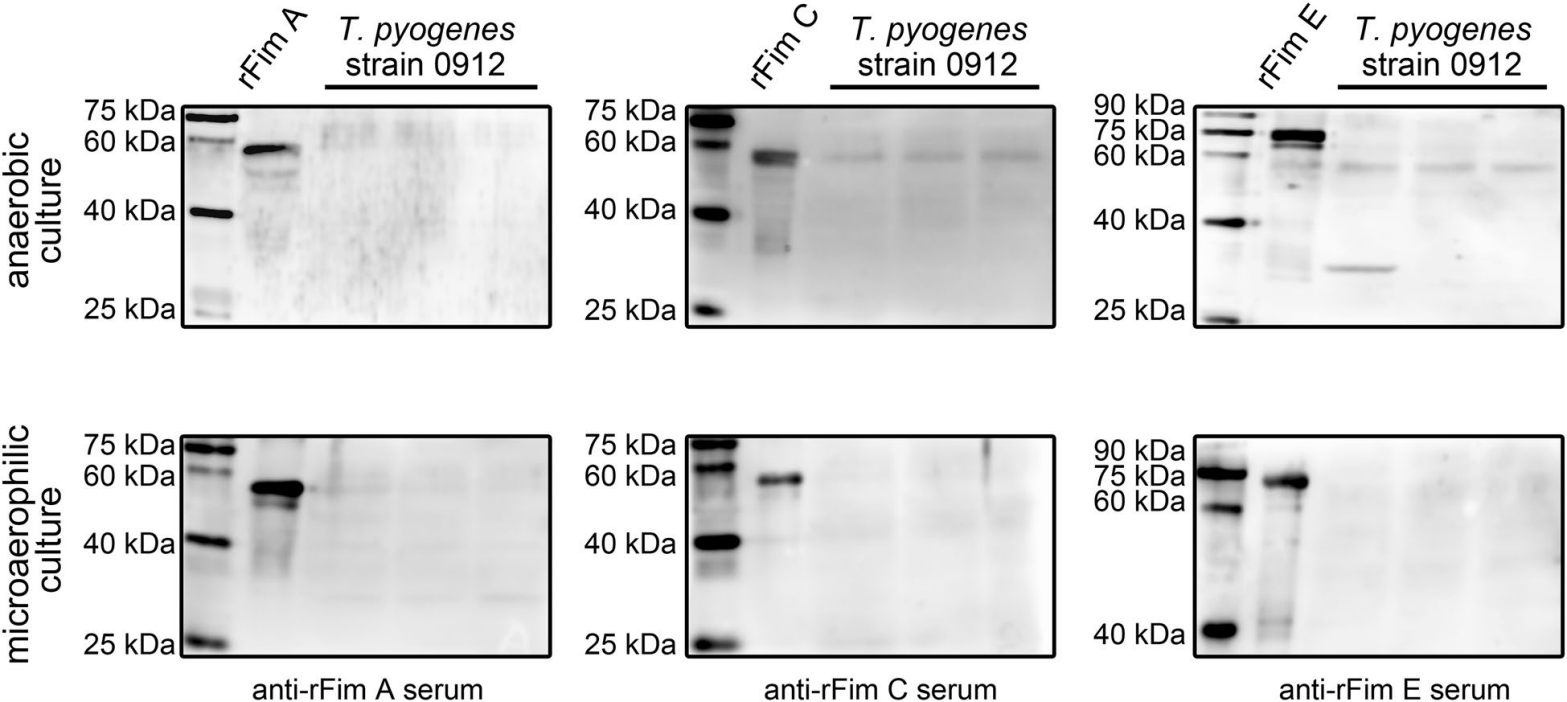
Analysis of the effects of anaerobic and microaerophilic environment on the expression of Fim A, Fim C, and Fim E in *T. pyogenes* strain 0912 by western blot assays. The samples for immunoblot assay were harvested from three different batches of cultured *T. pyogenes* strain 0912, cultured for 48 h in anaerobic environment or 24 h in microaerophilic environment

We finally tried to investigate the influence of anaerobic environment and microaerophilic environment on the expression of the three proteins in *T. pyogenes* strain 0912. Proliferation of the organism in anaerobic environments was not as efficient as proliferating in aerobic conditions. Figure 5 indicates that anaerobic environment did not affect the expression of the three recombinant proteins, and only the Fim E was detectable in the western blot assay. The microaerophilic environment improved the growth rate and promoted the production of pyolysin of the microorganisms on the plates (data not shown). However, Fim E was not detected in the microorganism by western blot assay using anti-rFim E serum, thus indicating that the microaerophilic environment could have inhibited the expression of Fim E (Fig. 5). Moreover, Fim A and Fim C were not detected in *T. pyogenes* cultured in the microaerophilic environment (Fig. 5).

### Tube agglutination test

**Fig. 6:**
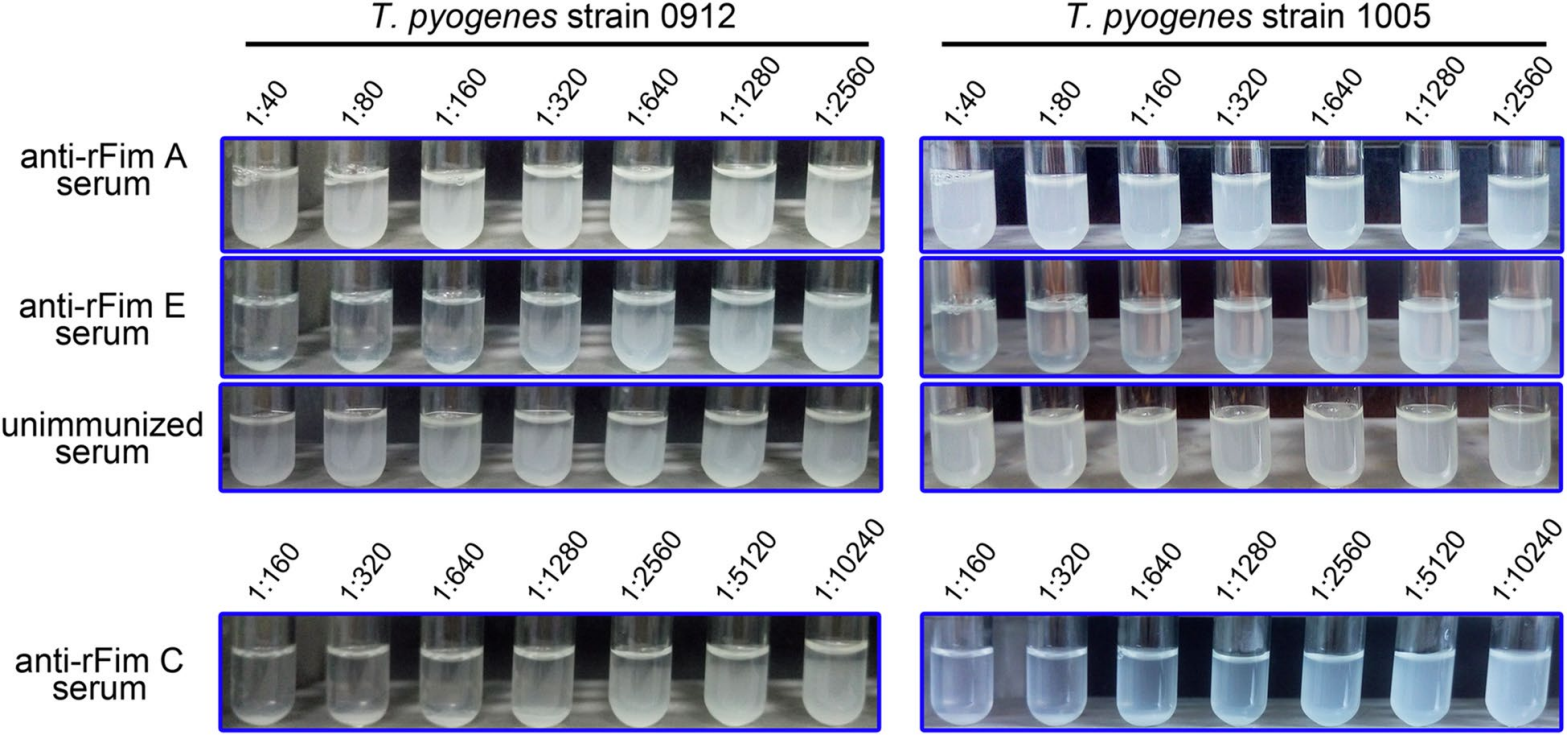
Analysis the reactivity of anti-rFim A, rFim C, and rFim E serum to intact *T. pyogenes* cells via tube agglutination test. *T. pyogenes* strain 0912 and strain 1005 cells were cultured for 8 h. Anti-rFim C and anti-rFim E serum caused agglutination of *T. pyogenes* strain 0912 and strain 1005 cells. Anti-rFim A serum failed to cause agglutination of *T. pyogenes* strain 0912 and strain 1005 cells. The experiment was repeated twice

Results of tube agglutination test showed that rabbit anti-rFim C and anti-rFim E sera caused noticeable agglutination of *T. pyogenes* strains 0912 and 1005 cells at dilution ratios as high as 1:1280 and 1:640 (Fig. 6), respectively. Results indicated that the antigens targeted by the rabbit anti-rFim C and anti-rFim E sera were exposed on the surface of the organism.

In contrast, rabbit anti-rFim A serum and the serum collected from an unimmunized rabbit did not cause noticeable agglutination of *T. pyogenes* cells within 30 min even at a dilution ratio of as low as 1:40 (Fig. 6).

## Discussion

The pathogenesis of infections caused by the opportunistic pathogen *T. pyogenes* remain poorly characterized. Opportunistic pathogenic infections take place when the host immune systems are affected by adverse factors, such as shipping, wounds, extreme weather, and primary pathogenic infections, that facilitate the colonization and invasion of these pathogens [15, 16]. In addition, the changes in expression profiles of the various virulence factors of pathogens might be essential in establishing infections. Jost and Billington [1] speculated that genetically regulated differential expression levels of virulence factors are among the reasons for the transformation of a commensal to a pathogenic organism in *T. pyogenes* infection. Thus, investigating the expression profiles of the virulence factors of *T. pyogenes* would help in comprehensively understand its pathogenesis. In the current study, the expression of three putative fimbriae proteins (Fim A, Fim C, and Fim E) in cultured *T. pyogenes* were investigated. The results showed that Fim E protein expression was evident; however, Fim A protein expression was not detected. Moreover, the expression of Fim E was inhibited by microaerophilic conditions.

We first determined the presence of Fim A-, Fim C-, and Fim E-specific antibodies in rabbit anti-*T. pyogenes* serum by immunizing rabbits with the bacterium. The results showed that only rFim E reacted to the anti-serum (Fig. 1), thereby indicating the lack of Fim A- and Fim C-specific antibodies in the serum. Formaldehyde treatment caused the overdenaturation of Fim A and Fim C, which might have caused the phenomenon. However, Fim E molecules in cultured *T. pyogenes* would also be denatured by the formaldehyde treatment and successfully elicited specific antibodies in rabbit. This implied that the molecules in the *T. pyogenes* were not overdenatured by the formaldehyde treatment. Additionally, the anti-*T. pyogenes* serum was prepared by immunizing the rabbit with formaldehyde-inactivated and live *T. pyogenes*. The protocol in the experiment proved the existence of antibodies that were specific to all the components of *T. pyogenes* cells in the serum. Hence, we assumed that the *fimA* and *fimC* genes were either not expressed or expressed in relatively low levels in cultured *T. pyogenes*, thereby leading to the failure of eliciting specific antibodies against the two proteins.

Then, we directly examined the expression of Fim A, Fim C, and Fim E in cultured *T. pyogenes*. Results in Figs. 2, 3, 4 indicate that only Fim E could be steadily detected in cultured *T. pyogenes* using western blot assay. The blots for Fim C detection showed strong nonspecific reaction bands (Figs. 2, 3, 4). Meanwhile, the anti-*T. pyogenes* serum failed to detect rFim C (Fig. 1). Thus, we could not draw a conclusion related to the expression profile of Fim C in cultured *T. pyogenes*; however, the anti-rFim C serum caused strong agglutination of *T. pyogenes*. Bisinotto [12] reported that anti-Fim C serum has been detected in cattle with *T. pyogenes* infection, and that the expression of Fim C by *T. pyogenes* is observable in vivo. To exactly determine the expression of Fim C in *T. pyogenes*, specific tools, such as monoclonal antibodies against Fim C, are needed. The expression of Fim A was not observed in *T. pyogenes* lysates using western blot assay (Figs. 2, 3, 4); meanwhile, the anti-rFim A serum did not cause agglutination in *T. pyogenes* within 30 min. Several immunoblots showed reactive components and similar molecular weights to the predicted molecular weight of Fim A in *T. pyogenes* lysates (*T. pyogenes* strain 1005 sampled at 16, 20, and 24 h in Fig. 3 and *T. pyogenes* strain 0912 cultured in pH 5.5 and 6.5 conditions in Fig. 4). Thus, we tested the reactivity of *T. pyogenes* cultured under these conditions to anti-rFim A serum via tube agglutination assay. Results showed no or only weak reaction between *T. pyogenes* and anti-rFim A serum (data not shown). These data indicated the Fim A was not, or poorly, expressed in cultured *T. pyogenes* in this experiment. The experiment exhibited two limitations: (1) only two isolated strains of *T. pyogenes* were involved and (2) a standard ATCC strain of *T. pyogenes* was lacking. Thus, further investigation is needed to determine whether the results of these experiments are universal in *T. pyogenes* strains, using more *T. pyogenes* strains isolated from different animal species and with different types of *T. pyogenes* infections.

Based on the results of the experiments, the microaerophilic condition promoted the proliferation of *T. pyogenes,* but inhibited the expression of Fim E in vitro. *Neisseria meningitidis* has been reported to posttranslationally modify one of its pili for the detachment of individual bacterium cells from the colony and colonizing new sites after several rounds of cell division while attached to a host cell [17]. This implies that the bacteria can regulate the expression of adhesins by controlling their attachment and detachment during infections. We speculated that the rapid proliferation of *T. pyogenes* in microaerophilic condition may spread *T. pyogenes* cells, thereby leading to the downregulation of adhesin protein expression. However, in the present study, the microaerophilic culture was only achieved when the organism was cultured on the plate. This led to another speculation that the expression of Fim E in *T. pyogenes* decreased due to the changes in the culturing method, but not because of the gaseous environment. This hypothesis should be addressed in future studies.

*T. pyogenes* has been reported to be less efficient to produce fimbriae under standard growth conditions [1]. Yanagawa and Honda [18] reported that only a small portion of cultured *T. pyogenes* have fimbriae (10%), and that the number of fimbriae on each piliated bacterial cell is less than 10. On the basis of our results, we speculated that the poor expression of fimbriae was at least partially attributed to the poor expression of Fim A, if the putative Fim A indeed participates in the assembly of *T. pyogenes* fimbriae.

Jost and Billington [1] and Bisinotto [12] reported the existence of antibodies against Fim A in *T. pyogenes*-infected cattle, thus indicating the expression of Fim A by *T. pyogenes* in vivo. Moreover, Bicalho [19] reported that the increased transcription of *fimA* gene of *T. pyogenes* is associated with the occurrence of metritis and endometritis in postpartum dairy cows. However, the results of Bisinotto’s study inferred the poor expression of Fim A by *T. pyogenes* compared with the expression levels of Fim C and Fim E, because the OD value of the ELISA for Fim A-specific antibody detection was noticeably lower than those of the ELISA for Fim C and Fim E-specific antibody detection [12].

An interesting phenomenon in Bisinotto’s report is that the titers of antibodies against Fim C and Fim E were associated with the presence of *T. pyogenes* in the uterus of cattle (constantly detectable if the *T. pyogenes* was detected in the cattle and consistently higher than that of herdmates without *T. pyogenes*), but were not significantly related to the day relative to calving. In contrast, the titers of antibodies against Fim A were not associated with the presence of *T. pyogenes*. However, the increase of the concentration of Fim A-specific antibodies was significantly related to the day relative to calving in cows with *T. pyogenes* [12]. Thus, we speculated that the expression of Fim C and Fim E by *T. pyogenes* in vivo are constitutive, whereas the expression of Fim A might be regulated by several factors related to the calving of the cattle.

Another possible mechanism related to the upregulation of Fim A expression in vivo might be a quorum-sensing response. Duarte [20] reported that the genome of *T. pyogenes* involves the *lsrACDBFGE* operon, which is related to the quorum-sensing signal response AI-2. Huang [21] reported that N-acyl homoserine lactones, a type of quorum-sensing molecule, significantly decreased the expression levels of virulence genes of residual *T. pyogenes* in a mouse model. These data infer the importance of quorum sensing in regulating the expression of virulence factors in *T. pyogenes*. Wagener [22] reported that the proportion of *T. pyogenes*-positive cows increased from 0.5% at day 0–85% at 15th day postpartum, as determined via traditional bacterial isolation method, thereby indicating the proliferation of *T. pyogenes* in the uterus of the cattle. Moreover, Williams [23] reported that the growth density of *T. pyogenes* from uterine lumen swabs collected from postpartum cows peaked in samples collected at the 14th day postpartum. The proliferation of the organism in the cattle uterus might raise the concentration of quorum-sensing molecules in tissues and upregulate the Fim A expression.

Combining the results of the previous and current studies, we speculated that the poor expression of Fim A restricts the formation of additional fimbriae by *T. pyogenes* in normal circumstances, and that *T. pyogenes* shows commensal characteristics. When the environmental conditions changed, the upregulation of Fim A expression promoted fimbriae assembly. Additional fimbriae and the upregulation of other virulence factor expressions facilitated the invasion of *T. pyogenes* into deep tissues. In future studies, genetic technology-mediated overexpression of Fim A protein in *T. pyogenes*, followed by the counting of fimbriae, might be a practical strategy to further study the role of Fim A in *T. pyogenes* infection.

## Conclusions

We determined the expression profile of the three putative fimbriae proteins, namely, Fim A, Fim C, and Fim E, in in vitro cultured *T. pyogenes*. Based on our results, only Fim E could be detected via western blot method. The expression of Fim E could be inhibited by microaerophilic condition. Our results would be helpful for comprehensively understanding the pathogenesis of *T. pyogenes* infections and developing *T. pyogenes* vaccines. The expression profile and role of Fim A and Fim C in *T. pyogenes* should be further studied, using more sensitive and specific tools and proper strategies.

## Additional file

**Additional file 1.** The amino acid sequences of the putative Fim A, Fim C and Fim E of *T. pyogenes* strain 0912. The amino acids in CWSS of each protein were colored by red.

## Abbreviations

aa: amino acids
bp: base pair
CWSS: cell wall sorting signal
NC: nitrocellulose
FBS: fetal bovine serum
HRP: horse radish peroxidase
IPTG: isopropyl-β-d-1-thiogalactoside
OD: optical density
PBS: phosphate buffer saline
rFim A: recombinant Fim A
rFim C: recombinant Fim C
rFim E: recombinant Fim E
SDS-PAGE: sodium dodecyl sulfate polyacrylamide gel electrophoresis
*T. pyogenes*: *Trueperella pyogenes*
