# Supplementary material for: Determination of the expression of three fimbrial subunit proteins in cultured Trueperella pyogenes
Source: Acta Vet Scand. 2018 Sep 12;60:53. doi: 10.1186/s13028-018-0407-3 (PMC6134790; doi:10.1186/s13028-018-0407-3)
Supplement: Supplementary file 1 — Additional file 1. The amino acid sequences of the putative Fim A, Fim C and Fim E of T. pyogenes strain 0912. The amino acids in CWSS of each protein were colored by red. [file 13028_2018_407_MOESM1_ESM.docx]

Additional file 1. The amino acid sequences of the putative Fim A, Fim C and Fim E of *T. pyogenes* strain 0912. The amino acids in CWSS of each protein were colored by red.

a. The amino acid sequence of Fim A of *T. pyogenes* strain 0912

1 MGALVTAFTL VSIGAATTAS

21 AKPSGFPVVD KQKATTLTIH

41 KHVGDEKFGK YVGEQRIEKD

61 PVVGVEFTVT PVLCELDLAT

81 PDAWKTISKA TVESASSCAN

101 RESKVVKTAE DGSAKIDLPQ

121 GIYKVEETKS GNNLVSTKSA

141 PFLVTLPMPK GDNEWVYDVH

161 AYPKNKLTEP GVPTKTASEP

181 TKFVPGAEIT WTVKASIPVL

201 QLPYESIVIT DQVPAGLTFK

221 EVTSVKIGNE TLSAGTDKAD

241 YSVANGVITL TPAGLEKVSS

261 AMAKASESMP VEVNLVTTVG

281 MDISTTGATT NKVTLTLNGK

301 ESKPGEGTTV WGNLIVNKVN

321 EKKELLDDAV FSIYAGKCEA

341 VTSGSTPVVE NLTTKGGVIT

361 QKLYVGKNES DSKDYCLKET

381 AAPAGYVLDP VGRTVTVKAG

401 KDATEIIEFE NVKVEGPD**LP**

421 **LTG**AQGTAIM VAAGLLLLAA

441 GAGTVYVARR RNA

b. The amino acid sequence of Fim C of *T. pyogenes* strain 0912

1 MSTRKVTRFG AVFTVVTLAS

21 LGATSAANAD TQSGFNAPDK

41 NHSVSLTVHK HEGNEKLDKY

61 TGQQQQPQGK AIAGVTFTVQ

81 EAGFDEGSGC KSIDLALPAS

101 WEKIAKAKPD SVCLLNDPVS

121 METNETGIAE FKQLSQKLYK

141 VTETAGGKNL IKTPSAPFLV

161 TLPMPVDPNK WVYDVHAYPK

181 NVLTELDDFT KKAADPADEK

201 GTKKFVPGAL ITWTIDATIP

221 KVAFDYTEVT MTDTVPAGLQ

241 FKAVKSVKLS GEPLAATQDY

261 TVTDSKIVLT EKGLAKLNPA

281 AKKNDVKVTV ELDTTVTDAI

301 LDGKTTNKVQ LSLNGKTKDA

321 NGDTYWGSIQ LTKQDKDAPN

341 TKLSDAVFSI YEGKCEANGA

361 VVAENLKTDQ QGVFKQKLYI

381 GNKEDATKDY CLKETAAPAG

401 YILDSTGIDF TLSVANDQFT

421 KNVTFDNVKV TGPH**LPLTG**A

441 QGTALLTGAG ILLLAVGAGT

461 VYHARRRS

c. The amino acid sequence of Fim E of *T. pyogenes* strain 0912

1 MKRNKLRAGA ALAVIALLGS

21 MGVGGLAHAA PAPKPQVTVA

41 DKDQSAANPA LIKKDATTQL

61 SIHKYLGTPV EAKNNGTIQK

81 IEDRTPLQNV QFDLYKVENV

101 DLTTNKGWEA AKALYERKVN

121 VADLNKGAIE IEGTNFTFSE

141 KTSGTTDASG AATIKAKVGL

161 YLVIENLSAS KDIKDGGKTY

181 TPAQITGINP FLVTLPMTNP

201 DSRDSWMYDV HVYPKNQAAE

221 MTKAVIDGNQ GKENQDGYKI

241 GQNITYRLES TINVVDSNQD

261 GKVDGDDLGY YLVKDQLSEH

281 VKYVSSKLSI IGSDNKTVEL

301 LTPQDYAFTN NSNLLSFSIT

321 KDGLNKLATA AGGKLQTEIV

341 TTVTTMPITG QVKNKASFHP

361 NNYPWTNSGK TPPKPGENPP

381 PGDTPPDVPS NEVVSKYGDV

401 VIKKINADKQ PLAGAEFAVF

421 RATKESDADG YTCKNVDFSK

441 APIAKTAAAS DAGGLTIVRG

461 LQLSNWRNDS SAKSGAITDE

481 KQFYSYCLVE TKSPDGYQLL

501 AEPIEFNLLK ESAVMDLSSS

521 EEAKMTDEVK KNGRALEVVN

541 QPDNLKNK**LP LTG**GEGIALV

561 SVLGILLVGG GAGYYIYANR

581 RKDV
